# The quality of web-based oncology guidelines and protocols: how do international sites stack up?

**DOI:** 10.1038/bjc.2011.378

**Published:** 2011-09-20

**Authors:** J M Langton, A K Drew, L Mellish, J Olivier, R L Ward, S-A Pearson

**Affiliations:** 1 Lowy Cancer Research Centre, Adult Cancer Program, Prince of Wales Clinical School, The University of New South Wales, Sydney, New South Wales, 2052, Australia

**Keywords:** medical oncology, chemotherapy, practice guideline, clinical protocol, decision making, computer assisted

## Abstract

**Background:**

The Internet is a popular medium for disseminating information relevant to oncology practitioners. Despite the widespread use of web-based guidelines and protocols, the quality of these resources has not been evaluated. This study addresses this gap.

**Methods:**

The Appraisal of Guidelines for Research and Evaluation (AGREE-II) instrument was used to assess the quality of breast and sarcoma guidelines and protocols according to six independent domains. The oncology resources were selected from eight websites developed for healthcare settings in North America, the United Kingdom, Europe, and Australia.

**Results:**

Mean quality scores across domains were highly variable for both guidelines (29–73%) and protocols (31–71%). Guidelines scored highly in terms of articulating their Scope and Purpose (72.6±11.2%) but poorly with respect to Applicability in clinical practice (29.0±17.3%). Protocols scored highly on Clarity of Presentation (70.6±17.6%) but poorly in terms of the processes used to synthesise underlying evidence, develop, and update recommendations (30.8±20.0%).

**Conclusion:**

Our evaluation provides a quick reference tool for clinicians about the strengths and limitations of oncology resources across several major websites. Further, it supports resource developers in terms of where to direct efforts to enhance guideline and protocol development processes or the communication of these processes to end-users.

In recent years there has been a significant shift in the way clinicians access practice-related resources. Medical oncology, like many other specialties, has embraced web-based technology. Research from the United States, Canada and Australia demonstrates that the Internet is being used increasingly as a major information source to support the treatment decisions of cancer clinicians, and to guide the delivery of care (Wong and Veness, 2005; Ousley *et al*, 2010). Several organisations have developed oncology websites, typically featuring clinical practice guidelines and/or treatment protocols, both of which synthesise the rapidly changing medical evidence-base. Given the increased awareness and use of web-based resources, there is a growing need to evaluate the quality of the information used in day-to-day oncology practice (Wong and Veness, 2005; Masters, 2008).

The literature abounds with evaluations of the ‘quality’ of web-based health information however, most research to date has focused primarily on content targeting patients (Kim *et al*, 1999; Eysenbach *et al*, 2002; Deshpande and Jadad, 2009). Further, there is no consensus amongst researchers about what constitutes a quality evaluation tool (Kim *et al*, 1999; Eysenbach *et al*, 2002; Gagliardi and Jadad, 2002; Carden *et al*, 2007). There are currently more than 50 instruments, comprising hundreds of criteria, being used to evaluate web-based health information. The paucity of research evaluating clinician-targeted websites is somewhat surprising, given their prolific use in clinical practice (Kuperman *et al*, 2001; Westbrook *et al*, 2004; Smith *et al*, 2006; Pearson *et al*, 2009). Therefore, the aim of this paper is to evaluate clinician-focused oncology websites. In the absence of a ‘gold standard’ instrument to compare website quality, we conducted a targeted assessment of specific oncology website content and features commonly used in clinical practice, namely guidelines and chemotherapy protocols.

The Appraisal of Guidelines for Research and Evaluation (AGREE) instrument (AGREE Next Steps Consortium, 2009) was designed and validated to evaluate the quality of clinical guidelines according to six independent domains. In the past decade, AGREE has been cited in over 150 peer-reviewed publications, and has been used to evaluate an extensive range of clinical guidelines across various areas of healthcare, including cancer (Harpole *et al*, 2003; Burgers *et al*, 2004). One previous evaluation, focusing predominantly on paper-based breast cancer guidelines, concluded oncology guidelines were of superior quality compared to guidelines developed for other medical specialties (Burgers *et al*, 2004). The AGREE has not been used previously to evaluate protocol quality, however, use in protocol development has been proposed as one of the several applications of the instrument (besides guideline assessment; Brouwers *et al*, 2010a). Indeed, the instrument's domains are equally applicable to protocol development and content. Therefore, in this paper we report on the quality of web-based guidelines and protocols for breast cancer and sarcoma. These cancers were chosen because of their contrasting prevalence rates and the differences in the evidence-base for the treatment of these diseases. We also report on a number of key features of the websites, and conduct a basic navigability assessment. This research provides clinicians with a quick reference guide on the strengths and limitations of oncology resources across several major websites.

## Materials and methods

### Website selection

To provide a meaningful commentary on the quality of web-based oncology resources, we consulted collaborators involved in cancer care and guideline/protocol development (RW–also a medical oncologist; and two external collaborators) to identify international websites disseminating resources that potentially satisfied the following criteria:

#### Website features

The website disseminated clinical guidelines and/or protocols. We defined guidelines as statements developed systematically to assist practitioner's decision-making in cancer care (Woolf *et al*, 1999), and defined protocols as evidence-based statements assisting practitioners in implementing treatment choices (specific information on implementing a particular chemotherapy protocol, e.g., dosing, scheduling, and monitoring). We did not evaluate sites containing only ‘information summaries’, which are evidence-based statements providing a general overview across various oncology topics (e.g., diagnosis, treatment).

#### Target audience

The website targeted health professionals. Sites could also disseminate patient-focused information; however, we excluded sites focused solely on patient information.

#### Scope

The website contained detailed information on more than one cancer type and more than one mode of cancer treatment.

Fourteen potential websites were identified by our collaborators and after reviewing them we found that eight websites satisfied the aforementioned criteria (Table 1). Websites were excluded for being patient-focused (*n*=3); not containing guidelines or protocols (*n*=1); and containing guidelines/protocols on only one aspect of cancer care (e.g., supportive care or screening only, *n*=2).

### Cancer selection

We evaluated guidelines and protocols for two cancers with different prevalence rates; breast cancer is the most prevalent cancer globally and sarcoma has prevalence rates of less than 1% (Parkin *et al*, 2005). Notably, these cancers also differ in the extent of the available treatment evidence-base; MEDLINE searches (15 February 2011) using mapped terms returned 124 548 articles for ‘breast cancer’ and 77 488 for ‘sarcoma’. In addition, our search of the US-based clinical trials registry (http://clinicaltrials.gov) revealed 2717 phase I, II and III breast cancer clinical trials, and 680 for sarcoma.

### Guideline/protocol selection

We selected one guideline and protocol for each cancer from the eight websites (if available). We aimed to evaluate resources that were as similar as possible; however, we did encounter some variability in the scope of resources across sites. We selected adjuvant breast cancer guidelines and chose a commonly administered adjuvant protocol common to all websites. For sarcoma, we chose guidelines and protocols covering soft tissue sarcoma. In the absence of this option, we selected an alternative guideline/protocol that was most similar to the other websites (this was the case for the National Institute of Clinical Excellence (NICE) sarcoma guideline and OncologySTAT sarcoma protocol).

### Appraisers

Four independent appraisers (JL, AD, LM, and SP; all healthcare professionals and/or researchers) pilot tested the AGREE-II instrument using six guidelines and protocols so as to become familiar with the tool and assess its appropriateness for rating protocols. Following pilot testing, three raters (AD, JL, and LM) evaluated all the resources presented in this paper. Rating procedures were conducted in accordance with the AGREE-II manual and were as follows:

- Appraisers independently rated up to five resources each week for 6 weeks (10 January 2011–18 February 2011).
- Scores were entered into a database weekly and discrepancies of >2 between scores of at least two raters were identified and re-rated, blind to the scores of other raters.
- A fourth reviewer (SP) rated items with outstanding disagreements.

### Instrument

AGREE was developed collaboratively by international researchers for use in guideline development and evaluation, and AGREE-II was launched in 2010 with improved validity and reliability, as well as a comprehensive users’ manual (AGREE Next Steps Consortium, 2009).

AGREE-II has 23 items grouped into six independent domains: Scope and Purpose, Stakeholder Involvement, Rigour of Development, Clarity of Presentation, Applicability, Editorial Independence, and a general question rating overall quality and whether appraisers recommend the guideline for use. Items are scored on a 7-point Likert scale (7=strongly agree; 1=strongly disagree). The AGREE-II has acceptable internal consistency (Cronbach's *α*=0.64–0.89), face, construct, and criterion validity (Brouwers *et al*, 2010b, 2010c).

### Reporting and analysis

#### Website characteristics

We documented website characteristics such as country of origin, site charges, advertising, and other key features. We also conducted a basic navigability assessment, defined as the number of pages (clicks) from the website homepage to reach a given guideline/protocol without using an internal search engine. Two independent raters (JL, AD) tallied the number of clicks to navigate to each of the protocols and guidelines evaluated. Scores were crosschecked; however, there were no discrepancies.

#### Guideline and protocol evaluation

We report the pattern of scores for guidelines and protocols across eight oncology websites. It was not our intent to hypothesis test or make direct comparisons between guideline and protocol quality. As detailed in the AGREE-II manual, we calculated a percentage score for each domain using the following formula; ((score obtained by raters−minimum score possible)/(maximum score possible−minimum score possible) × 100), such that the maximum achievable score is 100% and the minimum achievable score is 0%. On the basis of our pilot testing, we excluded question 18 from the protocol evaluation, as it was deemed not applicable. This item addresses the facilitators and barriers to applying recommendations; protocols by definition are about implementation after these factors have been considered and/or treatment decisions made. The AGREE-II manual recognises that some questions may not apply to all circumstances.

The AGREE-II does not provide guidance on what constitutes high *vs* low scores. Instead it advises that scores should be interpreted within the context of the project.

#### Inter-rater reliability

We assessed inter-rater reliability of the three assessors (JL, AD, and LM) using intra-class correlation coefficients (Streiner and Norman, 2003) according to the resource type (guidelines/protocols) and cancer type (breast/sarcoma). We also recalculated these statistics, based on the final scores, after disagreements were arbitrated by the fourth independent rater (SP).

## Results

### Website characteristics

We evaluated eight websites developed in North America, Europe, the United Kingdom, and Australia. All sites provided guideline access free of charge, with three websites charging additional fees for membership and/or access to other resources such as journal subscriptions. With the exception of National Comprehensive Cancer Network (NCCN), all websites provided access to protocols, free of charge. Only one website contained commercial advertising (OncologySTAT); however, most publicised relevant journals, clinical trials, and upcoming oncology conferences. The number of clicks required to navigate from the homepage to a given guideline/protocol ranged from two to five, suggesting resources were generally easy to find (Table 1).

### Guidelines

#### Domain scores

The combined mean domain scores for breast guidelines compared with combined sarcoma guidelines varied by a maximum of 10%. Therefore, we report the overall mean domain scores for breast and sarcoma guidelines combined (Table 2).

Scores across the six domains and general rating item were highly variable (range 29–73%). Average scores of greater than 50% were obtained in four of six domains plus the general rating item. Relative to other domains, Rigour of Development and Editorial Independence had the greatest range in scores (ranges of 5–85% and 2–92%, respectively). The Scope and Purpose domain scores were consistently high across all the websites (72.6±11.2%), with the Applicability domain scoring the lowest (29.0±17.3%). Overall, NICE breast and Cancer Care Ontario (CCO) sarcoma guidelines achieved the highest scores across most domains, with the British Columbia Cancer Agency (BCCA) guidelines scoring lowest across most domains. Guidelines scoring well in one domain generally scored well across all the domains.

Additionally, domain and overall rating scores for breast and sarcoma guidelines disseminated by the same website were generally consistent. Only 7 of 35 scores varied by more than 10%, and 4 of these were from NICE, in which the sarcoma guideline scored lower than the breast guideline.

#### Item scores

We attained mean scores of 5 or more (maximum score 7) on seven items: all questions in the Scope and Purpose domain (Items 1–3), clearly defining target users (Item 6) and all items in the Clarity of Presentation domain (Items 15–17). Scores of less than 3 were obtained for items referring to the process of seeking views and preferences of the target population, and providing descriptions of the strengths and limitations of the evidence informing the guidelines (Items 5, 9). Items relating to guideline implementation such as consideration of facilitators and barriers to application and resource implications of applying the recommendations also achieved scores of less than 3 (Items 18, 20) (Table 4).

### Protocols

#### Domain scores

The difference in mean domain scores for breast protocols combined and sarcoma protocols combined ranged from 1 to 6%. Therefore, we report the overall mean domain scores for breast and sarcoma protocols combined (Table 3).

There was a high degree of variability in the overall scores across the six domains and general rating item (31–71%). Average scores of greater than 50% were obtained for two of six domains, plus the general rating item. Relative to other domains, Rigour of Development and Editorial Independence had the greatest range in scores (10–68% and 0–89%, respectively). Clarity of Presentation was the highest scoring domain across protocols (70.6±17.6%); with the Rigour of Development score being the lowest (30.8±20.0%). Overall, CCO and eviQ protocols achieved the highest scores across most domains, with OncologySTAT protocols scoring the lowest across most domains for both breast and sarcoma. Protocols scoring well in one domain generally scored well across all domains.

We found little variation between domain scores for breast and sarcoma protocols disseminated by the same website. Only 3 of 35 scores yielded differences more than 10% with no difference of greater than 20%.

#### Item scores

Scores greater than 5 (maximum score 7) were obtained for two items in the Clarity of Presentation domain (providing recommendations that are specific and unambiguous; easily identifiable; Items 15, 17), and advice on putting recommendations into practice (Item 19). Scores of less than 3 were obtained for items addressing the process of seeking the views and preferences of the target population (Item 5), and five of the eight items in the Rigour of Development domain (Items 7–10, 13). Scores of less than 3 were also obtained for items such as consideration of the resource implications of applying the recommendations and recording/addressing the competing interests of development group members (Items 20, 23) (Table 4).

### Inter-rater reliability

The overall intra-class correlation coefficient was 0.80 before arbitration of discrepant scores by the fourth independent reviewer and 0.85 thereafter, suggesting the overall variance between raters was minimal. This was also the case when considering guidelines/protocols and breast/sarcoma cancers separately (range: 0.75–0.83). As expected, we found a noticeable improvement in the intra-class correlations after arbitration of discrepant scores (range: 0.84–0.85).

## Discussion

Our study is the first comprehensive evaluation of the quality of clinical guidelines and treatment protocols disseminated on websites designed for cancer clinicians. Using a psychometrically robust evaluation tool we identified areas of strength and deficiency, and distinguished between the quality of different oncology guidelines and protocols. Overall, the quality of oncology resources was modest.

In general, we found little variation between breast and sarcoma resources with respect to combined domain scores and for resources disseminated by the same website. This finding is not surprising, and suggests the processes underpinning the development of oncology resources do not vary by cancer type. We found the greatest variability in domains addressing the processes used to locate and summarise evidence, and to formulate and update recommendations. Further, items addressing the way in which bias and competing interests are minimised during development were highly variable. Our findings provide clear guidance to resource developers as to the way in which they can improve their processes and/or the way in which they are communicated to end-users. Clearly, collaboration across international boundaries would be prudent so as to minimise duplication of effort and costs of developing these tools (Brouwers *et al*, 2011). However, organisations must retain the capacity to tailor resources to the needs of the clinicians working in their own healthcare jurisdictions.

From the perspective of oncology practice, it is difficult to ascertain whether the differences demonstrated in resource quality will have meaningful effects on the uptake and use of oncology guidelines and protocols, or whether the use of higher quality resources equates to better quality care and improved outcomes for patients. The premise of oncology guidelines and protocols is to reduce treatment variation, adverse events, and patient mortality (Gandhi *et al*, 2005; Norton and Baker, 2007; Pavlidis *et al*, 2007; Manchon Walsh and Borras, 2009; Schwappach and Wernli, 2010). Research suggests the use of guidelines in oncology can reduce practice variation (Smith and Hillner, 2001; Ray-Coquard *et al*, 2005), yet there are very few examinations of the impact of oncology resources and computerised support systems on patient outcomes (Voeffray *et al*, 2006). Clearly, future research efforts should address this evidence void.

Systematic reviews in the area of prescriber behaviour change and computerised clinical support highlight repeatedly that the provision of clinical resources does not guarantee uptake (Kawamoto *et al*, 2005; Mollon *et al*, 2009; Moxey *et al*, 2010). Key factors critical to the success of computerised support systems include relevant content, having the ability to save limited clinical time, end-user involvement in development, and the ability of resources to be integrated into the clinical workflow. In the process of our evaluation, we noted that oncology websites typically fare well across these factors. All of the websites under review were geared to assist clinicians maintain an up-to-date and comprehensive knowledge of their field of practice (Grol and Grimshaw, 2003) by providing links to the oncology evidence-base, and updating their guidelines and/or protocols routinely (or at the very least providing dates of last update). Further, content development groups typically include end-users and resources were presented in a clear and accessible manner. Finally, given clinicians report their lack of time as a factor affecting the uptake of web-based tools (Bates *et al*, 2003; Hains *et al*, 2009, 2010), features such as clarity of presentation, quick reference summaries alongside guidelines, advice on implementing treatment (protocols), and easy-to-locate resources observed across the eight websites, suggests they are ideal for point-of-care use.

Our study is not without limitations. We acknowledge that our evaluation was not systematic, rather websites were selected by a small group of content experts and we evaluated a relatively small sample of guidelines and protocols. As such, our evaluation may not be representative of all oncology guidelines/protocols disseminated online. Further, our evaluation group is based in one healthcare setting and does not have members representing all of the websites under evaluation. Finally, we note that the AGREE-II was not developed to evaluate protocol quality. However, our initial psychometric evaluation demonstrated that the tool's properties were robust for this type of assessment, which may serve as preliminary evidence that the AGREE-II can be used for protocol assessment research.

Web-based information systems have revolutionised healthcare delivery and have the distinct advantage over paper-based systems because of their capacity to accommodate changes in best-practice evidence in real time. Given the increasing popularity of web-based tools in clinical practice, it is paramount we monitor and evaluate the quality of clinician-targeted resources disseminated online. It is unreasonable to expect individual practitioners to replicate the efforts detailed in this paper. As such we encourage continued evaluations of this kind, which provide a quick reference tool for oncology clinicians to scrutinise the resources they are using in their day-to-day practice. Further our research pinpoints where resource developers should direct their efforts to obtain high-quality guidelines and protocols.

## Figures and Tables

**Table 1:** Characteristics of the eight oncology websites included in our evaluation

**Website**	**Country of origin**	**Clinical guidelines?**	**Chemotherapy protocols?**	**Patient information or handouts?**	**Links to evidence base, for example, journal articles**	**Site charges**	**Advertising?**	**Login or registration required?**	**Minimum clicks to access guidelines (protocols)**
American Society of Clinical Oncology (ASCO) http://www.asco.org/portal/site/ascov2	USA	Yes	No	Links to www.cancer.net	Yes	Most guidelines and tools available for free. Membership fees to access JCO.	No	No	4 (n/a)
British Columbia Cancer Agency (BCCA) http://www.bccancer.bc.ca/default.htm	Canada	Yes	Yes	Yes	Yes	Free	No	No	2 (3)
Cancer Care Ontario (CCO) http://www.cancercare.on.ca/english/home/	Canada	Yes	Yes (regimens)	Yes	Yes	Free	No	No	3 (4)
European Society for Medical Oncology (ESMO) http://www.esmo.org/	Europe (mutli-national)	Yes	No	Yes	Yes	Access to clinical guidelines for free. Membership fees to access additional resources (e.g. drug calculators)	No	No	2 (n/a)
eviQ Cancer Treatments Online (Cancer Institute, NSW) http://www.eviq.org.au	Australia	No	Yes	Yes	Yes	Free	No	Yes	n/a (4)
National Comprehensive Cancer Network (NCCN) http://www.nccn.org/clinical.asp	USA	Yes	Yes (templates)	Links to www.NCCN.com	Yes	Access to clinical guidelines free. Annual fees to access chemotherapy templates (US$399) and other tools.	No	Yes	2 (2)
National Institute for Health and Clinical Excellence (NICE) http://www.nice.org.uk/	UK	Yes	No	Yes	Yes	Free	No	No	5 (n/a)
OncologySTAT http://www.oncologystat.com/index.html	USA	No	Yes (regimens)	No	Yes	Free	Yes (pharmaceutical)	Yes	n/a (2)

Abbreviations: n/a=not available; JCO=Journal of Clinical Oncology.

**Table 2:** Breast and sarcoma guideline domain scores, and mean combined scores from oncology websites

	**1. Scope and Purpose**	**2. Stakeholder Involvement**	**3. Rigour of Development**	**4. Clarity of Presentation**	**5. Applicability**	**6. Editorial Independence**	**General rating item**
*Breast cancer guidelines*							
ASCO	77.8	48.2	66.7	81.5	48.6	63.9	72.2
BCCA	57.4	29.6	11.8	57.4	23.6	2.8	44.4
CCO	**81.5**	64.8	67.4	72.2	13.9	**83.3**	66.7
ESMO	70.4	33.3	33.3	68.5	13.9	36.1	55.6
NICE	**81.5**	**85.2**	**84.7**	**92.6**	**61.1**	80.6	**94.4**
NCCN	61.1	59.3	42.4	75.9	20.8	91.7	66.7
*Breast combined*	71.6	53.4	51.0	74.7	30.3	59.7	66.7
							
*Sarcoma guidelines*							
ASCO	—	—	—	—	—	—	—
BCCA	51.9	27.8	4.9	55.6	29.2	2.8	44.4
CCO	**87.0**	59.3	**81.3**	**79.6**	11.1	80.6	**72.2**
ESMO	75.9	40.7	31.3	59.3	16.7	61.1	55.6
NICE	79.6	**79.6**	50.7	68.5	**52.8**	30.6	55.6
NCCN	74.1	53.7	39.6	72.2	27.8	**91.7**	66.7
*Sarcoma combined*	73.7	52.2	41.5	67.0	27.5	53.3	58.9
							
*Guidelines overall*							
Standard deviation	**72.6 (11.2)**	**52.9 (19.3)**	**46.7 (26.3)**	**71.2 (11.2)**	**29.0 (17.3)**	**58.8 (33.6)**	**63.1 (14.3)**
Range	**51–87**	**27–85**	**4–85**	**55–93**	**11–61**	**2–92**	**44–94**

Abbreviations: ASCO=American Society of Clinical Oncology; BCCA=British Columbia Cancer Agency; CCO=Cancer Care Ontario; ESMO=European Society for Medical Oncology; NICE=National Institute for Health and Clinical Excellence; NCCN=National Comprehensive Cancer Network.

Note: the highest performing guideline in each domain is in bold font; – refers to no data available.

**Table 3:** Breast and sarcoma protocol domain scores, and mean combined scores from oncology websites

	**1. Scope and Purpose**	**2. Stakeholder Involvement**	**3. Rigour of Development**	**4. Clarity of Presentation**	**5. Applicability**	**6. Editorial Independence**	**General rating item**
*Breast cancer protocols*							
BCCA	61.1	22.2	18.1	75.9	55.6	0.0	50.0
CCO	66.7	40.7	**68.1**	83.3	46.3	52.8	66.7
eviQ	**68.5**	33.3	36.8	**92.6**	**77.8**	19.4	**77.8**
NCCN	48.2	46.3	22.9	61.1	42.6	**83.3**	55.6
OncologySTAT	29.6	**48.2**	11.1	55.6	42.6	38.9	44.4
*Breast combined*	54.8	38.2	31.4	73.7	53.0	38.9	58.9
							
*Sarcoma protocols*							
BCCA	63.0	40.7	13.2	68.5	38.9	0.0	50.0
CCO	64.8	37.0	**59.0**	87.0	53.7	47.2	72.2
eviQ	**75.9**	35.2	37.5	**88.9**	**81.5**	25.0	**77.8**
NCCN	53.7	40.7	30.6	44.4	37.0	**88.9**	55.6
OncologySTAT	31.5	**44.4**	10.4	48.2	44.4	38.9	50.0
*Sarcoma combined*	57.8	39.6	30.1	67.4	51.1	40.0	61.1
							
*Protocols overall*							
Standard deviation	**56.3 (15.6)**	**38.9 (7.5)**	**30.8 (20.0)**	**70.6 (17.6)**	**47.8 (15.7)**	**36.4 (30.4)**	**60.0 (12.5)**
Range	**30–76**	**22–48**	**10–68**	**44–93**	**37–82**	**0–89**	**44–78**

Abbreviations: BCCA=British Columbia Cancer Agency; CCO=Cancer Care Ontario; NCCN=National Comprehensive Cancer Network.

Note: the highest performing guideline in each domain is in bold font.

**Table 4:** Mean item scores for oncology guidelines and protocols assessed with the AGREE-II instrument (maximum seven, minimum one)

**AGREE-II item**		**Guidelines (*n*=11), mean (s.d.)**	**Protocols (*n*=9), mean (s.d.)**
*Domain 1: Scope and Purpose*			
1	The overall objective(s) of the guideline is (are) specifically described.	5.3 (1.4)	4.3 (1.5)
2	The health question(s) covered by the guideline is (are) specifically described.	5.5 (1.1)	4.3 (1.1)
3	The population (patients, public, etc.) to whom the guideline is meant to apply is specifically described.	5.3 (1.1)	4.6 (1.2)
			
*Domain 2: Stakeholder Involvement*			
4	The guideline development group includes individuals from all relevant professional groups.	4.7 (1.3)	4.2 (1.4)
5	The views and preferences of the target population (patients, public, etc.) have been sought.	2.6 (2.0)	1.0 (0.2)
6	The target users of the guideline are clearly defined.	5.2 (1.7)	4.7 (0.8)
			
*Domain 3: Rigour of Development*			
7	Systematic methods were used to search for evidence.	3.7 (2.6)	2.7 (2.1)
8	The criteria for selecting the evidence are clearly described.	3.6 (2.4)	2.5 (1.9)
9	The strengths and limitations of the body of evidence are clearly described.	2.7 (1.8)	1.2 (0.5)
10	The methods for formulating the recommendations are clearly described.	3.4 (2.0)	2.4 (1.6)
11	The health benefits, side effects, and risks have been considered in formulating the recommendations.	4.0 (1.8)	4.3 (1.7)
12	There is an explicit link between the recommendations and the supporting evidence.	4.8 (2.0)	3.9 (1.9)
13	The guideline has been externally reviewed by experts before its publication.	4.3 (2.3)	2.4 (1.2)
14	A procedure for updating the guideline is provided.	4.1 (1.9)	3.4 (1.9)
			
*Domain 4: Clarity of Presentation*			
15	The recommendations are specific and unambiguous.	5.4 (1.1)	5.3 (1.2)
16	The different options for management of the condition or health issue are clearly presented.	5.1 (1.0)	4.7 (1.5)
17	Key recommendations are easily identifiable.	5.3 (1.2)	5.7 (1.1)
			
*Domain 5: Applicability*			
18	The guideline describes facilitators and barriers to its application.	2.2 (1.5)	N/A
19	The guideline provides advice and/or tools on how the recommendations can be put into practice.	3.2 (1.5)	5.3 (1.4)
20	The potential resource implications of applying the recommendations have been considered.	2.5 (2.2)	1.6 (1.1)
21	The guideline presents monitoring and/or auditing criteria.	3.1 (1.5)	4.5 (1.2)
			
*Domain 6: Editorial Independence*			
22	The views of the funding body have not influenced the content of the guideline.	4.4 (2.3)	4.2 (2.0)
23	Competing interests of guideline development group members have been recorded and addressed.	4.5 (2.3)	2.5 (2.1)
			
*General rating*			
	Rate the overall quality of this guideline.	4.8 (1.1)	4.6 (1.1)
